# Evaluating the impact of caloric restriction, body mass index and exercise on primary open-angle glaucoma: A review

**DOI:** 10.1177/11206721241274445

**Published:** 2024-08-22

**Authors:** Jonathan YM Lai, Patrick Mclarnon, Carl Sheridan, Neeru A Vallabh

**Affiliations:** 1 St. Paul's Eye Unit, 574314 Royal Liverpool University Hospital, Liverpool, UK; 2 Department of Eye and Vision Science, 105725 Institute of Life Course and Medical Sciences, University of Liverpool, Liverpool, UK

**Keywords:** Primary open-angle glaucoma, caloric restriction, body mass index, exercise

## Abstract

This literature review evaluates any possible links between primary open-angle glaucoma (POAG) and caloric restriction (CR), body mass index (BMI), and exercise, aiming to map the extent of the literature. Its primary objective is to recognise the nature and breadth of research evidence, identify possible gaps in these topics and develop future studies. The databases searched were MEDLINE (PudMed), Scopus and ScienceDirect, in April 2023 for articles published in English, with no date restriction. A total of 447 search results were retrieved. Of these, 73 were related to CR, 249 to BMI, and 125 to exercise. Records identified included systematic reviews, meta-analyses, randomised controlled trials, cohort studies and animal studies. CR has been shown to halt the degeneration of retinal ganglion cells and protect against various glaucomatous processes in animal models. Low BMI has been shown to be associated with an increased risk of POAG and a faster rate of visual field deterioration in POAG. However, the association between high BMI and POAG is not consistent. Exercise has been shown to cause mechanical, vascular, and neurobiological changes affecting the pathophysiology of POAG. The present review helps identify key characteristics and factors relating to the impacts of CR, BMI, or exercise on POAG.

## Introduction

Glaucoma is the second leading cause of global blindness after cataracts, estimated to affect 76 million people in 2020 and 111.8 million by 2040 worldwide.^1^ Glaucoma is a term referring to a group of optic neuropathies characterised by retinal ganglion cell (RGC) degeneration, a distinctive loss of retinal nerve fibres and optic disc changes, which can lead to specific patterns of progressive visual field loss, usually beginning at the paracentral region.^2^

Primary open-angle glaucoma (POAG) is the predominant subtype of glaucoma and will be the main focus of this review. Open-angle refers to the anterior chamber angle, where the aqueous humour is primarily drained, is macroscopically open. It has been estimated that of all patients with glaucoma, 74% will have POAG.^3^ The aetiology of POAG is unclear, but it is thought to be an interplay between genetic and environmental factors. The main risk factors for POAG are age, high myopia, a positive family history of glaucoma, and elevated intraocular pressure (IOP) (>21 mm Hg).^4^

Pathophysiologically, POAG has been linked to an increased resistance to aqueous outflow through the trabecular meshwork (TM). The TM is the contractile tissue responsible for draining most of the aqueous humour from the anterior chamber of the eye. The specific mechanisms by which TM fails to maintain normal outflow resistance, leading to increased IOP, have been associated with several processes, such as the thickening of the elastic fibre sheaths, decreased cell density and remodelling of the extracellular matrix.^5^ A rise in IOP can cause direct and ischaemic damage to the RGC axons, and is currently the only modifiable risk factor for POAG. It has been shown that lowering elevated IOP can reduce the risk of POAG development^6^ and deter its progression.^7^ Lowering IOP remains the mainstay of POAG treatment, which can be achieved by reducing aqueous humour production and increasing aqueous humour outflow through regular eyedrops, laser therapy, surgery, or combinations.

However, glaucomatous changes in the optic nerve can also be seen in patients with normal IOP, termed normal tension glaucoma. In the Japanese population, it was found that 92% of patients with POAG have an IOP of 21 mmHg or less.^8^ It has been hypothesised that neuroinflammation,^9–12^ vascular changes,^13–15^ and mitochondrial dysfunction^16–18^ may also play a role in the pathogenesis of POAG or glaucoma. Other factors that influence glaucoma have also been described in the literature.

This literature review evaluates any possible links between glaucoma and caloric restriction (CR), body mass index (BMI), and exercise, aiming to map the extent of the literature. Its primary objective is to recognise the nature and breadth of research evidence, identify possible gaps in these topics and develop future studies.

## Methods

The databases searched were MEDLINE (PudMed), Scopus and ScienceDirect, in April 2023 for articles published in English, with no date restriction. Three separate searches were conducted: the following terms were used: (1) ‘(glaucoma) OR (primary open-angle glaucoma) AND (caloric restriction); (2) ‘(glaucoma) OR (primary open-angle glaucoma) AND (body mass index); and (3) ‘(glaucoma) OR (primary open-angle glaucoma) AND (exercise)’. All studies were considered, including systemic reviews, meta-analyses, randomised or non-randomised clinical trials, observational and experimental studies. Book chapters, case reports, policy papers, and letters were excluded to contain the search and screening.

## Study selection

All titles and abstracts were screened. Studies were included in the review if they mentioned CR, BMI or exercise in the context of POAG. Where it was uncertain, studies were included for full-text review.

## Data extraction and analysis

Data from selected studies was extracted using Microsoft Excel following recommended methods for scoping review.^19^ Findings were grouped by study design, population, intervention, comparison and outcome. Relevant in-article references not returned in our searches were also considered. The results for each subtopic were accompanied by narrative and thematic summaries describing how they relate to the review objectives.

## Results

**Figure 1.**
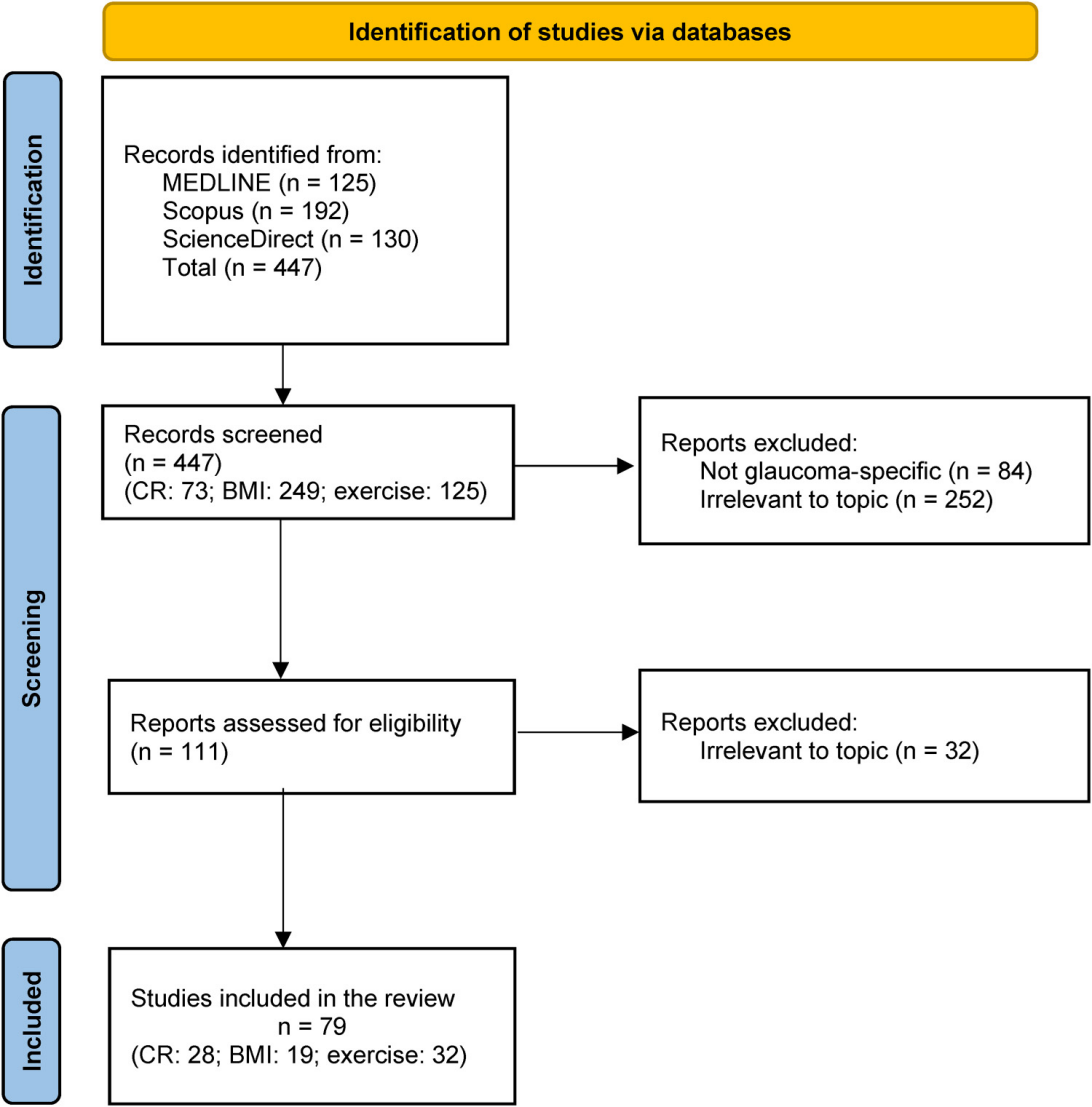
Study selection. Preferred Reporting Items for Systematic Reviews and Meta-analyses for Scoping Reviews (PRISMA-ScR) flow diagram.

CR: Caloric restriction; BMI: Body mass index

A total of 447 search results were retrieved. Of these, 73 were related to CR, 249 to BMI, and 125 to exercise. Titles and abstracts were screened by a single reviewer. One hundred and eleven records were selected for full-text review. Seventy-nine (CR: 28; BMI: 19; exercise: 32) were included in the review. For CR, there were nine reviews, 13 animal studies, four randomised clinical trials (RCTs) and two cohort studies; for BMI, five reviews, 10 cohort and four cross-sectional studies; for exercise, eight reviews, five cohort, six cross-sectional, nine RCT, and four animal studies. The results and study inclusion/exclusion process are reported and presented in Figure 1.

## Caloric restriction and glaucoma

Caloric restriction (CR) is the reduction of average calorie intake without deprivation of essential nutrients. Various regimens of CR exist depending on time and length. CR has been shown to extend lifespans in a wide variety of species and delay progression in animal models of neurodegenerative conditions.^20^ Neurodegenerative diseases, including Alzheimer's disease (AD), Parkinson's disease (PD) and Huntington's disease, are characterised by neuronal loss, which can be initiated by oxidative injury, glutamate excitotoxicity and the accumulation of protein aggregates.^21^ On a molecular level, CR has been shown to halt neurodegeneration by activating the mammalian target of rapamycin (mTOR) and adenosine monophosphate-activated kinase (AMPK) pathways, reducing reactive oxygen species (ROS)/oxidative stress, and modulating epigenetic factors.^22^ On a cellular level, CR is associated with higher synaptic plasticity and stimulation of neuroprotective pathways in the brain, leading to improved cognition.^23^

Glaucoma involves a neurodegenerative process where the RGCs malfunction over time and eventually die. CR has been proposed to halt the neurodegenerative process of RGCs and have a neuroprotective effect against insults.^24^ No human studies have examined the relationship between total caloric intake and glaucoma. A 10-year cohort study (183,067 person-years) proposed that higher total carbohydrate intake, regardless of the food source, is associated with a higher risk of glaucoma after adjusting for smoking, caffeine and alcohol intake, weight, height and medical history such as diabetes and cardiovascular disease (hazard ratio [HR] 1.5; 95% confidence interval [CI] 1.01–2.25).^25^ However, the HR shows a weak association, and the study was limited by its cohort, which consisted of only young middle-aged university graduates (average age of 37.8). Furthermore, the carbohydrate intake was assessed only at the baseline of the study and not monitored throughout the follow-up period. The incidence of glaucoma in this study was self-reported without systematic ophthalmological examinations, and ocular risk factors such as IOP were not adjusted.

## Caloric restriction and retinal function

Whilst there are no direct studies of CR and retinal function in humans, work on animal models has allowed more controlled studies. One such animal model of Brown Norway rats study examined the effect of a 40% reduction in caloric intake on retinal ageing.^26^ The retina of the CR rats was shown to increase cell densities and height in the outer nuclear layer, the peripheral inner nuclear layer of the retina, and the total retinal thickness compared to an ad libitum diet in the same rat model. Another group investigated the effect of every-other-day fasting (EODF) in EAAC1-/- (excitatory amino acid carrier 1-deficient) normal-tension glaucoma mouse model,^27^ and the results suggested that RGC death and retinal degeneration were suppressed in the EODF group, independent of IOP measurements. The vision had also improved with EODF on multifocal electroretinogram (mfERG). Furthermore, CR has been shown to be protective against loss of RGCs in the peripheral retina following ischaemia-reperfusion retinal damage^28^ and improve retinal functional recovery following an IOP-induced injury in rats.^29^

## Caloric restriction and neuroprotection

In a study that investigated the protein profile of the ageing neural retina of Brown Norway rats, CR was shown to maintain or stimulate proteins that are essential in geroprotective signalling pathways and halting age-related retinal degenerations.^30^ These proteins are mainly glycolytic enzymes and molecular chaperones, including phosphoglycerate kinase 1 (PGK1), glyceraldehyde 3-phosphate dehydrogenase (GAPDH), and two malate dehydrogenases (MDHs). Further, the neuroprotective effects of CR have been proposed to be linked with increased β-hydroxybutyrate (β-HB) levels and histone acetylation in the retina.^27^ β-HB involves cellular signalling and gene and protein expression. An increased β-HB level has been linked to a reduction in metabolic complications caused by insulin resistance and cellular ageing phenotypes, including senescence and inflammation.^31^ In addition, CR is thought to prevent mitochondrial dysfunction, which has been implicated in glaucomatous pathogenesis and progression.^17,18,32–35^ CR has been demonstrated to reduce oxidative stress following injury and improve mitochondrial oxidative phosphorylation compared with ad libitum controls.^29^ It is thought that enhanced mitochondrial function may increase energy availability in RGCs for repair processes and preventing apoptosis.^18^

## Caloric restriction and aqueous humour outflow

Ageing and oxidative stress have been shown to cause TM dysfunction by facilitating apoptosis of TM cells and extracellular matrix remodelling.^36^ Cell senescence in TM has been proposed to drive further tissue fibrosis and stiffening.^37^ These mechanisms can affect the aqueous outflow pathway and are accelerated in POAG.^38,39^ CR has been shown to prevent oxidative insults and limit senescent cell accumulation via modulating mTOR, insulin-like growth factor (IGF), mitochondrial sirtuin (SIRT), autophagy and DNA repair pathways.^40^ There have been limited studies on the direct effect of CR on aqueous humour outflow. However, Li and Wolf specifically addressed outflow in CR mice eyes. Interestingly, the aqueous collector channel size of the outflow pathway is reduced (30% reduction at 30–35 months of age (*p* < 0.01)) with age-related glaucomatous changes. The team found that the aqueous collecting channel's width and lumen area in CR mice remained normal compared to ad libitum-fed mice by 35 months of age.^41^ This may maintain adequate aqueous humour drainage, leading to better IOP control and consequently halting the development of POAG.

## Caloric restriction mimics

Metformin hasbeen shown to lower the risk of neurodegenerative diseases in animal models, including AD and PD.^42^ It has been shown to have a transcriptional profile similar to CR in mice.^43^ A prospective cohort study (*n* = 11,260) showed that patients with type 2 diabetes who were treated with metformin were associated with a lower risk of POAG (odd ratio [OR] 0.18; 95% CI 0.08–0.41; *p* < 0.001).^44^ One retrospective cohort study of patients with diabetes (*n* = 150,016) has shown that for every 1 g increase in metformin use, there is associated 0.16% reduction in POAG risk (adjusted HR 0.99984; 95% CI 0.99969–0.99999; *p* = 0.04), which predicts that taking a standard dose of 2 g of metformin per day for two years would result in a 20.8% reduction in POAG risk, independent of glycaemic control.^45^ However, no studies have evaluated metformin's effect on POAG in the non-diabetic group.^46^

Metformin has been proposed to act directly and indirectly on AMPK, proliferator-activated receptor γ (PPARγ), and mammalian target of rapamycin complex 1 (mTORC1), affecting survival, stress-defence, autophagy, oxidative stress, and protein synthesis. This, in turn, has anti-inflammatory, anti-fibrotic, anti-angiogenic, and anti-ageing effects.^47,48^ Metformin has been shown to have both beneficial and detrimental effects on mitochondrial functions. It inhibits Complex I and permeability transition pores (PTP) in the respiratory chain. The inhibition of Complex I would produce ROS and lead to apoptosis, whereas the inhibition of PTP will prevent this process.^48^ The mechanism of action of metformin is complex, and its effect on POAG requires further studies.

**Figure 2.**
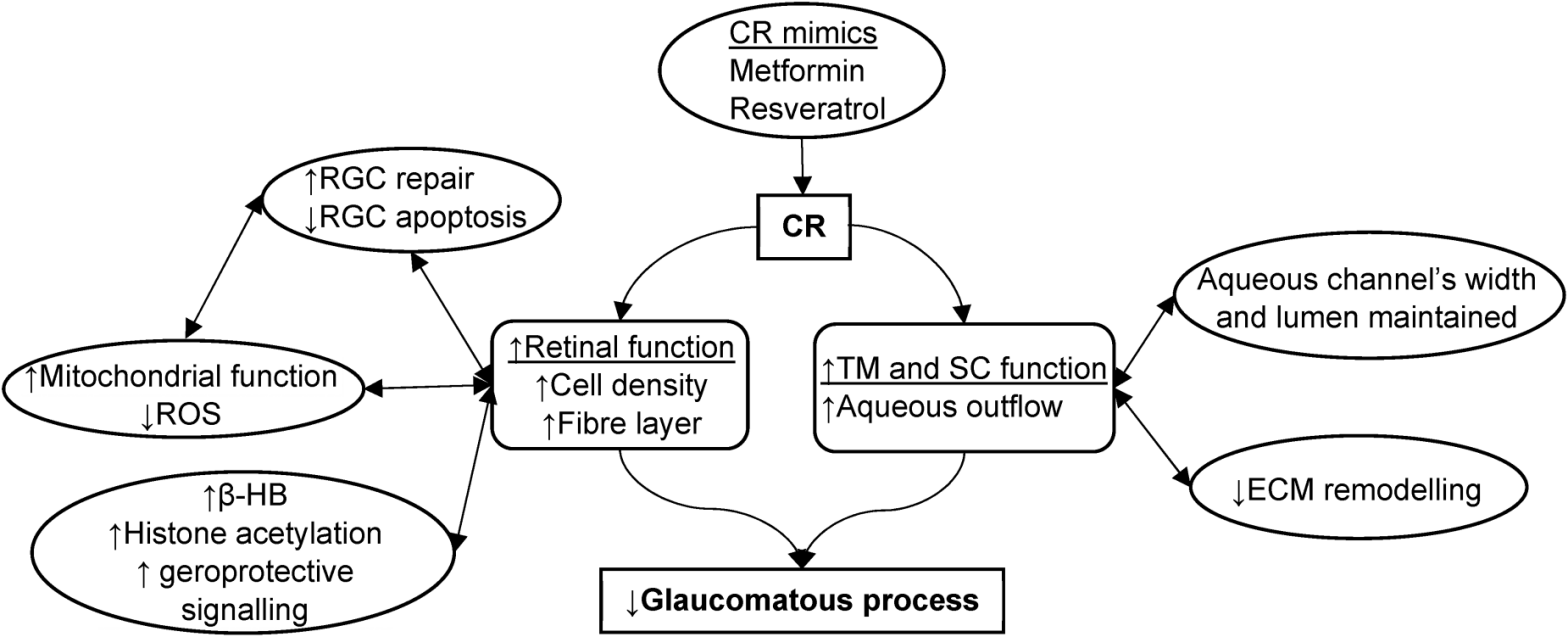
A figure illustrating hypothesised mechanisms of caloric restriction on glaucomatous pathophysiology.

CR: Caloric restriction; RGC: Retinal ganglion cell; β-HB: β-Hydroxybutyric acid; TM: Trabecular meshwork; SC: Schlemm's canal; ROS: Reactive oxygen species; ECM: Extracellular matrix.

Resveratrol, another CR mimic, has also been studied due to its ability to suppress ageing-related phenotypes by inhibiting cAMP phosphodiesterases (PDEs).^49^ A review of in vitro studies has discussed its effects on neuroprotection in RGCs. Resveratrol decreases the level of an apoptotic signalling molecule, cleaves caspase-3 and upregulates nuclear-encoded respiratory complex protein (NRF1) and mitochondrial transcription factor A (TFAM). On a cellular level, it stimulates mitochondrial biogenesis and prevents the loss of RGCs.^50^ Similar results were also demonstrated in a rat chronic ocular hypertension model, where resveratrol restored protein levels that were reduced in POAG via enhancing mitochondrial transcription and replication.^51^ Furthermore, resveratrol has been shown to reduce inflammatory markers in glaucomatous human TM cells by inducing antioxidant nitric oxide (NO) production.^49^ It also prevents the expression of glaucoma markers – senescence-associated beta-galactosidase (SA-β-gal), lipofuscin – and the accumulation of carbonylated proteins induced by chronic oxidative stress in TM cells.^52^ However, the clinical significance of resveratrol in POAG is currently unknown. Figure 2 summarises the hypothesised mechanisms of CR on glaucomatous pathophysiology.

## Body mass index and glaucoma

Body mass index (BMI) is a statistical index of nutritional status in adults, an estimate of body fat using a person's height and weight. According to the World Health Organisation, a BMI of 25 kg/m^2^ or above is classified as overweight, above 30 kg/m^2^ is obese, and a BMI below <18.5 kg/m^2^ is underweight. A cross-sectional study (*n* = 10,978) showed that lower BMI (<19 kg/m^2^) was associated with a greater risk of POAG compared with normal BMI (19–24.9 kg/m^2^) (OR 2.28; 95% CI 1.22–4.26).^53^ A multicohort observational study (UK and Canada) has shown that a lower BMI confers a 10% greater likelihood of POAG diagnosis (OR 0.90; 95% CI 0.84–0.98), a faster rate of visual field deterioration (β 0.04 dB/year/SD; 95% CI 0.01–0.07), and a worse cross-sectional vertical cup-disc ratio (β −0.05/SD; 95% CI 0.06–0.96).^54^

A 3-year retrospective cohort study (*n* = 17,000,636) in the South Korean population has demonstrated that being underweight (BMI <18.5 kg/m^2^) increases the risk of future POAG development by 9.8% (HR 1.10; 95% CI 1.07–1.13) in individuals without diabetes and 27.8% (HR 1.28; 95% CI 1.21–1.35) with diabetes. In contrast, the risk of POAG decreases in obesity class I (BMI 25.0–29.9 kg/m^2^) by 2.2% (HR 0.98; 95% CI 0.97–1.00) and in obesity class II (BMI ≥30 kg/m^2^) by 3.9% (HR 0.96; 95% CI 0.93–0.99) among individuals without diabetes; decrease in obesity class II by 19.5% (HR 0.81; 95% CI 0.79–0.82) and class II 22.8 (HR 0.77; 95% CI 0.74–0.80) among individuals with diabetes.^55^ The POAG risk reduction associated with obesity is more significant in the diabetic group in the study. A more recent prospective cohort study (*n* = 13,357) in China has shown that BMI ≥30 is associated with a 10.2% decrease in POAG risk based on the Cox proportional hazards model (HR 0.90; 95% CI 0.83–0.97) and an 11.8% risk reduction in the multivariate logistic regression analysis (OR 0.88; 95% CI 0.80–0.97).^56^ However, this study was self-reported and relied primarily on patients’ own knowledge of their condition.

Contradictorily, a cross-sectional study (*n* = 15,585) in South Korea has shown that a BMI ≥30 is more likely to develop POAG than those with a normal BMI, and it also positively correlates with glaucomatous neuropathy based on the optic cup-to-disc ratios (OR 1.27; 95% CI 1.07–1.51).^57^ Similar results were also found in a two-database matched-cohort study in the Taiwan population (adjusted HR 1.54; 95% CI 1.23–1.94).^58^

## Body mass index and retinal nerve fibre layer

A thinning of the retinal nerve fibre layer (RNFL) – 73–89 µm or below – is associated with visual field loss in POAG.^59^ A study (*n* = 2595) found that higher BMI is associated with a long-term reduction in ganglion cell fibre layers. It also negatively correlated with the thickness of RNFL/ganglion cell-inner plexiform (GCIPL) in men (*p* = 0.001/<0.001), and GCIPL in women (*p* = 0.03).^60^ Excluding people who have raised IOP, a cross-sectional study (*n* = 139) has found that RNFL thickness was reduced significantly in the superior nasal (107.8 ± 19.5 vs. 114.6 ± 22.4 μm (β 0.20, *p* = 0.01)) and superior temporal (135.7 ± 18.9 vs. 140.7 ± 18.2 μm (β 0.14, *p* = 0.05)) sections in people with metabolic syndrome compared than those without, which was independent of age.^61^ The mechanism by which obesity and metabolic syndrome are linked to RNFL thinning is unclear. It has been proposed that vascular risk associated with a high BMI (e.g., hypertension, dyslipidaemia, diabetes and increased clotting) may be a factor.^60^

The inconsistent association between the risk of POAG and BMI might be owing to varied study designs, populations, diagnostic criteria and confounding factors such as IOP. A cohort study (*n* = 196) found that high BMI positively correlates with high IOP, and the mean average IOP is 15.5 ± 2.5 mmHg in morbidly obese (≥40 kg/m^2^) and 14.5 ± 2.6 mmHg in normal BMIs (18.5–24.99 kg/m^2^).^62^ Obesity has been shown to cause increased blood viscosity, leading to increased outflow resistance in the episcleral veins and increasing IOP.^63^ Interestingly, a prospective cohort study (*n* = 32) found that one year following bariatric surgery, the mean IOP decreased by 16.7% (a mean of 2.8 mmHg) 3–6 months after the procedure, independently of the extent of BMI reduction.^64^ The results suggest that the relationship between BMI and IOP might not be linear and complex. However, no causal link has been established in these studies. It is also noteworthy that BMI has been shown to be a poor indicator of body fat percentage as it does not differentiate between lean muscle and fat mass.^65^ Furthermore, a prospective cohort study in the South Korean population (*n* = 287,553) revealed that individuals who are metabolically unhealthy (i.e., have at least one of diabetes mellitus, hypertension, and hypercholesterolaemia) have an increased risk of developing POAG (i.e., metabolically unhealthy and obese: adjusted HR, 1.57; 95% CI, 1.45–1.71; metabolically unhealthy and not obese: adjusted HR, 1.52; 95% CI 1.41–1.65), regardless of whether they are obese or of normal BMI (i.e., metabolically healthy and obese: adjusted HR, 1.02; 95% CI 0.91–1.14).^66^ The finding suggests that metabolic health rather than obesity may be a more specific measure of POAG risk.

## Exercise and glaucoma

In a systemic review, physical activity has been demonstrated to be inversely correlated with the risk of dementia in AD and PD.^67^ A clinical trial has shown that high-intensity treadmill exercise improved motor symptoms of patients with PD.^68^ Exercise has been shown to delay cognitive decline in neurodegenerative diseases by reducing chronic inflammation and metabolic disturbances, enhancing angiogenesis and synaptic plasticity.^69^ It has been proposed that exercise stimulates brain-derived neurotrophic factor (BDNF), a key molecule for promoting neuronal survival and synaptic plasticity.^70^ Exercise also stimulates vascular growth factors and increases cerebral blood flow.^71^

In POAG, a prospective study (*n* = 9519, mean follow-up of 5.7 years) showed that the incidence risk is reduced in active individuals (≥500 metabolic equivalent task [MET]/min/week, which equates to the American Heart Association's recommendation of 150 min of moderate-intensity aerobic exercise each week for optimal cardiovascular health) compared to inactive individuals (0 MET/min/week) (HR 0.6; 95% CI 0.38–0.95).^72^ In a cross-sectional study (*n* = 1387), people who spent the day standing or walking had a reduced risk of POAG compared to sitting (OR 0.42; 95% CI 0.25–0.70), and those who performed moderate-intensity activity had a significantly reduced risk compared with low-intensity activity based on accelerometers (OR 0.05; 95% CI 0.01–0.56).^73^ Both studies were self-reported, and a causal relationship between exercise levels and POAG risk could not be established.

## Exercise and intraocular pressure

Dynamic exercise has been shown to induce a transient reduction of IOP in healthy individuals and patients with POAG.^74–76^ A review has concluded that the reduction in IOP correlates with the intensity of the exercise but less with the duration of exercise, independently of anti-glaucoma agents use.^77^ The mechanism of lowering IOP following exercise has been proposed to be related to increased plasma colloid osmotic pressure.^78^ During or after dynamic exercise, fluid shifts from plasma to skeletal muscle interstitium. The effect may cause ocular dehydration, lowering the IOP. It has also been proposed that altered colloid pressure may act directly on the hypothalamus, activating a reflex response that leads to IOP changes.^79^ In contrast, activities that involve Valsalva manoeuvres with increased expiratory effort, such as lifting tasks, are associated with an elevation of IOP^80^ and a reduction in ocular perfusion.^81^ The elevation in IOP during Valsalva manoeuvres has been linked to raised episcleral pressure by an engorged anterior choroidal vessel, a narrowed anterior chamber, and higher resistance of the aqueous humour.^82^

A study (*n* = 20) found that common yoga positions that involve isometric exercise increase IOP within two minutes after returning to a sitting position in both healthy and individuals with glaucoma: downward dog pose (from 17 ± 3.2 mmHg to 28 ± 3.8 mmHg), standing forward bend (17 ± 3.9 mmHg to 27 ± 3.4 mmHg), plough pose (18 ± 2.8 mmHg to 24 ± 3.5 mmHg) and legs up the wall pose (17 ± 4 mmHg to 21 ± 3.6 mmHg).^83^ Yoga that involves a head-down position has been thought to increase episcleral venous pressure and choroidal thickness due to increased intracranial cerebrospinal fluid pressure. This may influence the choroid veins that drain into the superior ophthalmic vein and intracranial cavernous sinus, potentially increasing the IOP.^84^ However, yoga positions that involve meditation, gazing steadily at a point, or breathing exercises have been shown to reduce IOP. A meta-analysis of three RCTs involving 212 patients has shown that 4–6 weeks of the yoga exercises mentioned above can reduce mean IOP on the glaucomatous eye by 1.4 mmHg (average of both eyes; 95% CI 0.17–2.82; I^2^ = 76%), compared to glaucoma-free eyes.^85^

For the long-term effects of exercise on IOP, one cohort study in the Japanese population (*n* = 3119; mean age of 60) has shown a mean 5-year IOP reduction of 0.84 ± 1.9 mmHg in individuals without glaucoma and that the degree of IOP reduction is associated with increased frequency and increased exercise time (*p* < 0.05).^86^ A Malaysian interventional study (*n* = 45; mean age of 33) has shown similar results, a mean IOP reduction of 2.18 ± 2.25 after six weeks of regular exercise (*p* < 0.001).^87^ However, these studies were based on POAG-free young, healthy individuals and relied on their self-reporting. Furthermore, the exercise intensity was not defined. Overall, the degree of long-term IOP changes with dynamic exercise is difficult to interpret because the exercise intensity was measured differently between studies. The mechanism of IOP reduction through long-term exercise has been thought to be linked to cardiovascular factors such as systolic blood pressure and BMI.^77^

## Exercise and ocular perfusion pressure/ocular blood flow

Ocular perfusion pressure (OPP) refers to the net pressure gradient causing ocular blood flow (OBF) to the eye, driving oxygenated blood into the eye tissues, including the optic nerve. The blood vessels in the macular area are thinner, with less elasticity and fewer vascular regulating factors, rendering the surrounding tissues a higher risk of ischaemic damage. Ocular blood flow reduces with decreasing perfusion pressure, especially in POAG patients with impaired vascular autoregulation.^88^ Low OPP has been proposed as an important risk factor for POAG development and progression.^46,89,90^ Dynamic exercise increases OBF^74^ and OPP in individuals with POAG,^75,91^ which may help protect RGCs from ischaemia.^92^

Exercise-induced OBF changes have been shown to be mediated by nitric oxide (NO) and endothelin-1 (ET-1). The NO metabolite levels in the eyes increased immediately after dynamic exercise, which correlates to increased blood flow in the retina for 45 min measured by Doppler velocimetry and laser speckle flowgraphy, and a decrease in IOP that persists for an hour.^93^ It has been proposed that NO induces several vasodilatory responses in ocular vessels via acetylcholine, bradykinin and histamine,^94^ which leads to the relaxation of ophthalmic and ciliary arteries.^95^ Further, it has been shown that NO relaxes TM cells and reduces their cellular volume via potassium channel, increasing aqueous humour outflow.^96,97^ Impaired NO production has been implicated with a higher risk of glaucoma.^98^

ET-1 has been shown to play a role in choroidal vasoconstriction and an increased OPP during isometric exercise.^99^ ET-1 is a vasoconstrictor produced by the vascular endothelium, the retinal epithelium, and the ciliary epithelium.^100^ ET-1 has been shown to cause contraction of the trabecular meshwork and increased IOP, vasoconstriction, and reduced OBF, leading to RGC degeneration.^101^ Elevated plasma ET-1 level has been associated with glaucoma progression.^102^ ET-1 may also have a direct apoptotic effect on RGCs and play a role in anterograde axonal transport in the optic nerve.^101^ It has been proposed that ET-1 level is a risk factor for a biomarker for ocular blood flow changes in the optic nerve head of glaucomatous eyes.^103^

## Exercise and neuroprotection

Exercise can decrease key mechanisms associated with progressive neurodegeneration, such as mitochondrial dysfunction, oxidative stress and inflammation.^104^ In aged mice, exercise by swimming was shown to reverse the IOP-induced optic nerve damage measured by ERG. The study also demonstrated that exercise reduces the stress response in retinal glia, macrophages, and neurons.^105^ Another mice study showed that exercise prevented RGC and inner retina synaptic loss after IOP-induced injury.^106^ However, both experimental studies generated optic nerve damage using short-term elevation of IOP, which is different from chronic and degenerative pathological processes seen in POAG.

The neuroprotective effect of exercise was found to be dependent on BDNF. The inhibition of BNDF has been shown to diminish RGC protection induced by exercise.^107,108^ Blocking BNDF signalling with ANA-12 abolished the exercise-induced functional protection of RGC by preventing the elimination of synapses in the retina of a mouse model.^106^ Synapse remodelling with abnormal dendritic branching patterns has been reported in animal models of glaucoma prior to RGC death.^109^ The molecular mechanisms driving synaptic remodelling in glaucoma or injury are unknown. However, complement-mediated synaptic elimination may be involved. Complement proteins bind and tag defective CNS synapses for pruning, and this process may be aberrantly activated during neuroinflammation and neurodegenerative processes.^110^ Exercise after RGC injury in mice has been shown to inhibit synaptic deposition of complement,^106^ suggesting its role in neuroprotection.

**Figure 3.**
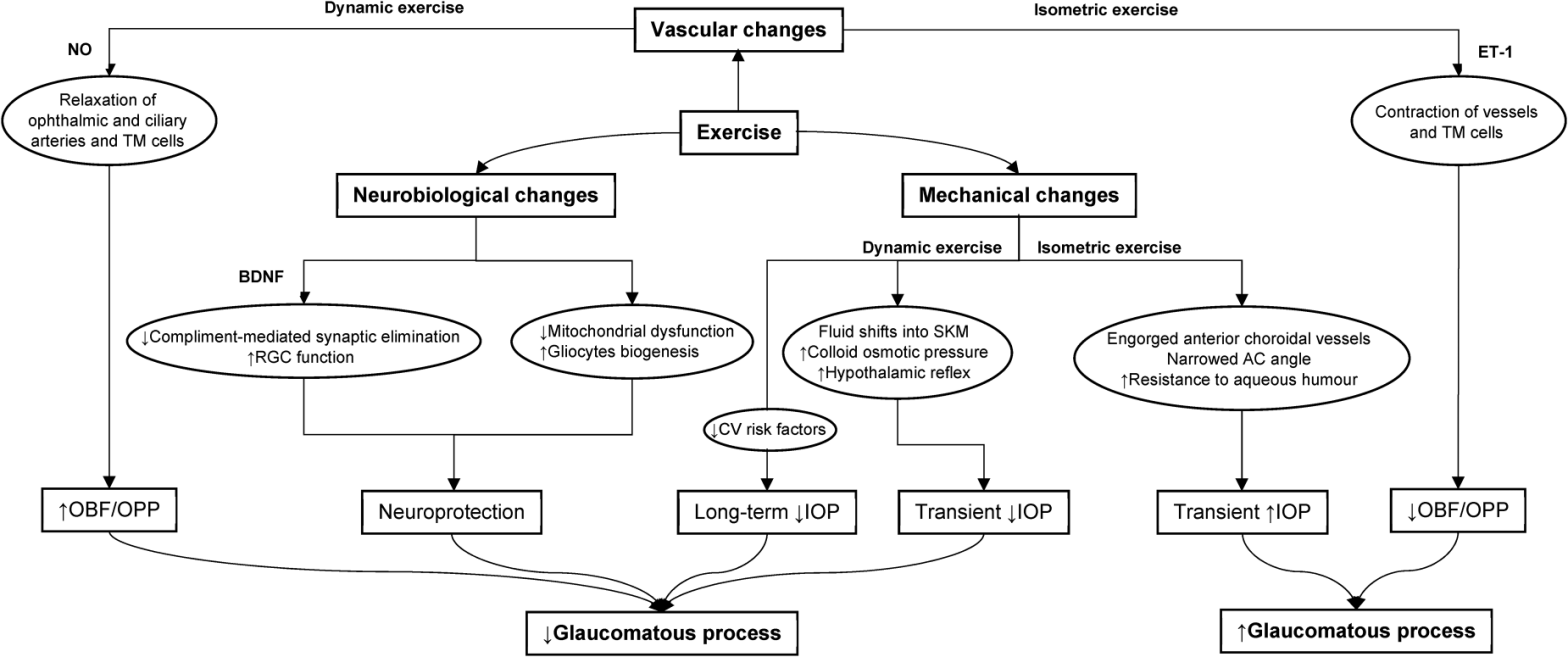
A figure illustrating hypothesised mechanisms of exercise on glaucomatous pathophysiology.

NO: Nitric oxide; ET-1: Endothelin-1; TM: Trabecular meshwork; BDNF: Brain-derived neurotrophic factor; RGC: Retinal ganglion cell; SKM: Skeletal muscle; AC: Anterior chamber; CV: Cardiovascular; OBF: Ocular blood flow; OPP: Ocular perfusion pressure; IOP: Intraocular pressure

In addition, exercise has also been shown to reverse hypoxia-induced mitochondrial dysfunction by lowering the mitochondrial membrane potential and elevating the matrix oxidant burden of lymphocytes.^111^ Exercise-induced lactate has also been shown to help promote the function and survival of Müller cells (retinal gliocytes that provide trophic and anti-oxidative support), indirectly protecting the RGCs.^112^ The hypothesised effects of exercise on glaucomatous pathophysiology are summarised in Figure 3.

## Conclusion

Caloric restriction (CR) studies are limited to animal models and have no direct association with POAG. BMI studies are mostly observational; the results are restricted to binary outcomes i.e., either high or low BMIs, and they are inconsistent. While exercise studies are based on humans, they focus primarily on the physiological parameters and biochemical factors related to POAG rather than in the clinical context.

CR has been shown to halt the degeneration of RGCs and protect against various glaucomatous processes in animal models. The neuroprotective effects of CR have been proposed to be linked with some geroprotective proteins, increased β-HB levels, histone acetylation and mitochondrial function in the retina. CR may also inhibit the oxidative changes in TM cells seen in ageing and POAG, reducing the aqueous humour flow resistance and halting the glaucomatous insult to the optic nerve. Therapies that mimic CR may represent new clinical treatments in POAG. Metformin has been shown to reduce POAG risk, but the studies have only included people with diabetes.

Low BMI has been shown to be associated with an increased risk of POAG and a faster rate of visual field deterioration in POAG. However, the association between high BMI and POAG is not consistent. High BMIs are linked with thinning of RNFL, potentially through endothelial and/or inflammatory changes. Further research is required to determine how high BMI affects IOP and the degree of RNFL thinning in the context of POAG. Furthermore, evidence suggests that metabolic health rather than BMI may be a more specific predictor of POAG risk.

Exercise has been shown to cause mechanical, vascular, and neurobiological changes affecting the pathophysiology of POAG. Isometric exercise transiently increases IOP, whereas dynamic exercise transiently reduces IOP. Additionally, yoga exercises that involve a head-down position increase IOP briefly, and others that involve meditation, gazing steadily at a point, or breathing exercises may lower IOP. Dynamic exercise has been demonstrated to reduce IOP in the long term, possibly by reducing cardiovascular risk factors. Dynamic exercise is also theorised to increase OBF and OPP via NO, whereas isometric exercise decreases OBF and OPP through ET-1. In addition, dynamic exercise has been proposed to have neuroprotective effects on RGCs, such as preventing synaptic elimination via BDNF, halting mitochondrial dysfunction, and reducing oxidative stress and inflammation.

The present review identifies key characteristics and factors relating to the impacts of CR, BMI, and exercise on POAG. However, it does not provide conclusive evidence, and further research is required. The risk of POAG onset or progression over time may be challenging to measure because of the concurrent treatments and the fact that caloric intake, BMI, and exercise may be interlinked. Designing clinical trials to investigate these three factors is difficult for the same reasons, in addition to the inability to control unknown confounding factors and blinding. Whilst clinical trials are challenging, discovery science unlocking the pathobiology and pathophysiology of healthy ageing and disease will yield therapeutic targets to complement lifestyle interventions.
